# Effects of ginkgo diterpene lactone glucosamine combined with clopidogrel on hemodynamics, neurocytokines, and inflammatory responses in patients with cerebral infarction complicated by coronary heart disease

**DOI:** 10.5937/jomb0-57275

**Published:** 2025-10-28

**Authors:** Shengjiao Zhu, Guoqiang Chen

**Affiliations:** 1 Huanggang Central Hospital, Department of Laboratory Medicine, Huanggang, Hubei, 438000, China

**Keywords:** ginkgo diterpene lactone meglumine, clopidogrel, cerebral infarction, coronary heart disease, hemodynamics, neurocytokines, inflammatory response, ginko diterpen lakton meglumin, klopidogrel, cerebralni infarkt, koronarna bolest srca, hemodinamika, neurocitokini, inflamatorni odgovor

## Abstract

**Background:**

This study investigates the effects of Ginkgo Diterpene Lactone Meglumine (GM) combined with Clopidogrel (CLO) on hemodynamics, neurocytokines, and inflammatory responses in patients with cerebral infarction (CI) complicated by coronary heart disease (CHD).

**Methods:**

A total of 152 patients diagnosed with CI complicated by CHD, admitted to our hospital between January 2024 and October 2024, were enrolled in the study. Among them, 81 patients received CLO monotherapy (control group), while the remaining 71 patients were treated with a combination of CLO and GM (observation group). Hemodynamic parameters, including plasma viscosity (PV), whole blood high (WBHSV) and low shear viscosity (WBLSV), as well as reduced viscosity (RV), were measured before and after treatment. Platelet adhesion test (PAdT) and platelet aggregation test (PAgT) were also performed. Inflammatory markers and neurocytokines were assessed using enzyme-linked immunosorbent assays, and adverse reactions during treatment were documented.

**Results:**

After treatment, both groups exhibited significant reductions in PAdT, PAgT, PV, WBHSV, WBLSV, and RV compared to baseline (P&lt;0.05). However, PAdT, PAgT, WBHSV, WBLSV and RV were lower in the observation group compared to the control group (P&lt;0.05). Additionally, the observation group showed lower levels of neuron-specific enolase, glial fibrillary acidic protein, tumor necrosis factor-a, and hypersensitive C-reactive protein, along with higher levels of brain-derived neurotrophic factor, compared to the control group (P&lt;0.05). No significant difference was observed in the incidence of adverse reactions between the two groups (P&gt;0.05).

**Conclusions:**

The combination of GM and CLO is more effective than CLO monotherapy in improving hemodynamics, enhancing neurological function, and mitigating inflammatory responses in patients with CI complicated by CHD.

## Abstract (sr)

**Uvod:**

Ova studija istražuje efekte Ginkgo Diterpene Lactone Meglumine (GM) u kombinaciji sa klopidogrelom (CLO) na hemodinamiku, neurocitokine i inflamatorne odgovore kod pacijenata sa cerebralnim infarktom (CI) komplikovanim koronarnom bolešću srca (CHD).

**Metode:**

U studiju je uključeno ukupno 152 pacijenta sa dijagnozom CI komplikovane koronarnom bolešću, koji su primljeni u našu bolnicu između januara 2024. i oktobra 2024. godine. Među njima, 81 pacijent je primio monoterapiju CLO (kontrolna grupa), dok je preostalih 71 pacijent lečen kombinacijom CLO i GM (grupa posmatranja). Hemo dinamski parametri, uključujući viskozitet plazme (PV), visok (VBHSV) i niski viskozitet na smicanje (VBLSV), kao i smanjeni viskozitet (RV), mereni su pre i posle tret-mana. Urađeni su i test adhezije trombocita (PAdT) i test agregacije trombocita (PAgT). Inflamatorni markeri i neu-rocitokini su procenjeni korišćenjem enzimskih imuno -sorbentnih testova, a neželjene reakcije tokom lečenja su dokumentovane.

**Rezultati:**

Nakon tretmana, obe grupe su pokazale značajno smanjenje PAdT , PAgT , PV , VBHSV , VBLSV i RV u poređenju sa početnom linijom (P&lt;0,05). Međutim, PAdT , PAgT , VBHSV , VBLSV i RV su bili niži u posmatranoj grupi u poređenju sa kontrolnom grupom (P&lt;0,05). Pored toga, posmatračka grupa je pokazala niže nivoe neuron-specifične enolaze, glijalnog fibrilarnog kiselog proteina, fakto-ra tumorske nekroze-a i preosetljivog C-reaktivnog proteina, zajedno sa višim nivoima neurotrofnog faktora koji potiče iz mozga, u poređenju sa kontrolnom grupom (P&lt;0,05). Nije primećena značajna razlika u incidenci neželjenih reakcija između dve grupe (P&gt;0,05).

**Zaključak:**

Kombinacija GM i CLO je efikasnija od mono -terapije CLO u poboljšanju hemodinamike, poboljšanju neuroloških funkcija i ublažavanju inflamatornih odgovora kod pacijenata sa CI komplikovanom CHD.

## Introduction

Cardiovascular and cerebrovascular diseases remain among the leading causes of global mortality.Epidemiological data indicate that the prevalence of these diseases is approximately 724 per 100,000 individuals [1]. Notably, the associated burden has risen significantly, with the number of deaths attributed to cardiovascular diseases and cerebrovascular diseases increasing markedly from 13.406 million and 4.503 million in 1990 to 17.267 million and 9.487 million in 2010, respectively [2]. Coronary heart disease (CHD) and cerebral infarction (CI), two hallmark conditions of cardiovascular and cerebrovascular diseases, share a common pathogenesis related to atherosclerotic plaque formation, often leading to their co-occurrence [3]. Studies reveal that approximately 20–30% of CI patients also present with CHD [4]. Current clinical management strategies emphasize the protection and repair of vascular endothelial cells, plaque stabilization, and the inhibition of coagulation, oxidation, and thrombosis [5]. Among these, clopidogrel (CLO) is one of the most widely utilized therapeutic agents [6]. However, long-term CLO use is associated with limited efficacy, frequent adverse reactions, and the development of drug resistance [7]. Therefore, optimizing the treatment for patients with CI and CHD has become a critical focus in modern clinical research.

In recent years, traditional Chinese medicine (TCM) has garnered increasing attention in the management of cardiovascular and cerebrovascular diseases due to its favorable safety profile and consistent therapeutic effects. Ginkgo Diterpene Lactone Meglumine (GM), a proprietary Chinese medicine composed of the active components of Ginkgo biloba, is recognized for its ability to enhance blood circulation, re solve blood stasis, and improve meridian function [8]. In CI treatment, a meta-analysis by Li J et al. [9] de mon strated the efficacy and safety of GM. Furthermore, recent pharmacological studies by Li Y et al. [10] highlighted the cardioprotective effects of GM in cardiovascular diseases, including CHD, providing a scientific basis for its potential application in CI complicated by CHD.

Despite these promising findings, clinical evidence validating the efficacy of this combination therapy in this specific patient population remains scarce. Against this background, the aim of this study was to evaluate the therapeutic effect of GM combined with CLO in patients with CI combined with CHD, and to further investigate in depth the changes in hemodynamics, neurocytokines and inflammatory factors before and after treatment, so as to determine the clinical value of GM combined with CLO as a treatment option and to provide new insights and guidance for the treatment of this complex condition.

## Materials and methods

### Study subjects

**Figure 1:**
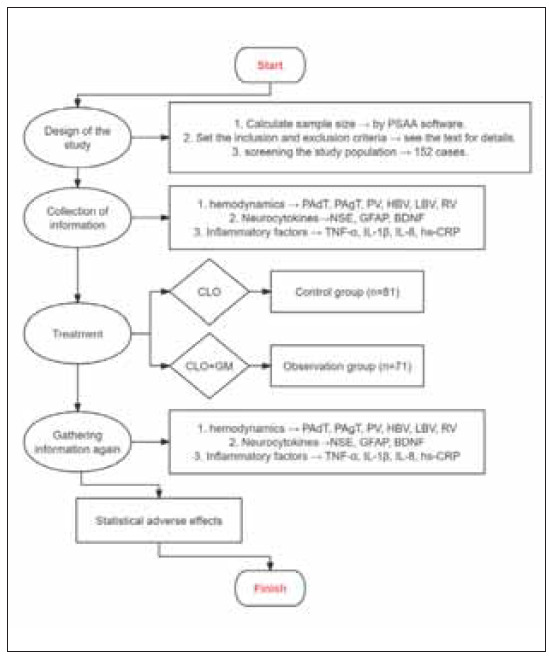
Flow of this study.

This retrospective study included 152 patients diagnosed with both CI and CHD who were admitted to our hospital between January 2024 and October 2024. All patients received CLO as part of their treatment regimen. Among them, 71 patients were additionally administered GM and assigned to the observation group, while the remaining 81 patients served as the control group. All patients and data collectors were unaware of their subgroups. The study workflow is presented in Figure 1. The study protocol was approved by the Ethics Committee of our hospital (NO. HGYY-KY-2025-001) and conducted in compliance with the principles outlined in the Declaration of Helsinki. Written informed consent was obtained from all participants prior to their inclusion in the study.

### Inclusion and exclusion criteria

Inclusion criteria: (1) Diagnosis of CHD [11] and CI [12] confirmed by clinical examination. (2) Age 60 years. (3) Presentation beyond the thrombolytic therapy time window (4.5h). (4) Normal cognitive function.

Exclusion criteria: (1) Presence of autoimmune diseases. (2) Coagulation disorders. (3) Impaired function of other major organs. (4) Diagnosis of malignant tumors. (5) Comorbid conditions such asmyocarditis, rheumatic heart disease, or cardiomyopathy. (6) History of brain surgery. (7) Known allergy to any of the medications used in this study.

### Treatment protocol

Upon admission, all patients were treated with CLO (CSPC Pharmaceutical Group Limited, Ou Yi Pharmaceutical Co., Ltd., H20193160), administered as one tablet once daily for 14 consecutive days. In addition, the patients were given oral aspirin (Shenyang Aojina Pharmaceutical Co., Ltd, H20065051), 100 mg/d, 1 time/d. Patients in the observation group additionally received GM (Jiangsu MyKanion Biological Medicine Co., Ltd., Z20120024) via intravenous infusion. GM was administered at a dose of 5 mL, diluted in 250 mL of 0.9% sodium chloride injection, once daily. The initial infusion rate was set at 15 drops per minute, and if no significant adverse reactions were observed within 30 minutes, the rate was increased to 40 drops per minute. The treatment duration was also 14 consecutive days.

### Laboratory assessments

Fasting venous blood was collected from patients before and after treatment and divided into 2 portions. Blood rheology parameters, including plasma viscosity (PV), whole blood high shear viscosity (WBHSV), whole blood low shear viscosity (WBLSV), and reduced viscosity (RV), were measured using a blood rheology analyzer (HT-100A, Zibo Hengtuo Analytical Instrument Co., Ltd.). Platelet adhesion test (PAdT) and platelet aggregation test (PAgT) were also conducted. Additionally, serum levels of tumor necrosis factor-α (TNF-α), interleukin-1β (IL-1β), interleukin-8 (IL-8), hypersensitive C-reactive protein (hs-CRP), neuron-specific enolase (NSE), glial fibrillary acidic protein (GFAP), and brain-derived neurotrophic factor (BDNF) were quantified using enzyme-linked immunosorbent assays (ELISA). The kits were purchased from Wuhan Fion Bio-technology Co. Ltd, and the operation process was carried out in strict accordance with the instructions of the kits.

### Outcome measures

The primary outcomes included changes in hemodynamic parameters (PV, WBHSV, WBLSV, RV, PAdT, and PAgT), neurocytokine levels (NSE, GFAP, and BDNF), and inflammatory markers (TNF-α, IL-1β, IL-8, and hs-CRP) before and after treatment in both groups. Secondary outcomes included the incidence of adverse reactions during the treatment period.

### Statistical analysis

Data analysis was performed using SPSS 26.0 software. Categorical variables, expressed as percentages (%), were compared using the chi-square test. For continuous variables, the Shapiro-Wilk test was used to assess data distribution. Normally distributed data, presented as mean ± standard deviation, were analyzed using independent t-tests for between-group comparisons and paired t-tests for within-group comparisons. Non-normally distributed data, expressed as median (interquartile range), were analyzed using the Mann-Whitney U test for between-group comparisons and the Wilcoxon signed-rank test for within-group comparisons. For the simultaneous analysis of multiple indicators, the Benjamini-Hochberg (BH) method was used to control for the false discovery rate (FDR) and to calculate corrected q-values. A P-value<0.05 was considered statistically significant.

## Results

### Baseline characteristics were comparable between groups

A comparison of baseline characteristics, including age, gender, and disease duration, revealed no statistically significant differences between the observation group and the control group (*P*>0.05, Table 1). This confirmed that the two groups were well-matched and suitable for comparative analysis.

**Table 1:** Comparison of clinical data. There was no statistically significant difference between the two groups. Note: Duration of disease (h) refers to the time from the onset of the patient’s illness until admission to the hospital. CHD combined with CI is the onset of CHD followed by CI; the reverse is true for CI combined with CHD.

Projects	Control group<br>(n=81)	Observation group<br>(n=71)	*t* or *χ^2^*-values	*P*-values
Age	66.06±3.38	66.65±4.09	0.968	0.335
Male	49 (60.49%)	37 (52.11%)	1.082	0.298
Female	32 (39.51%)	34 (47.89%)		
Duration of disease (h)	16.07±2.78	15.82±2.82	0.565	0.573
Body mass index (kg/m^2^)	22.73±1.93	22.45±1.72	0.956	0.341
Type of disease combination			0.220	0.639
CHD combined with CI	53 (65.43%)	49 (69.01%)		
CI combined with CHD	28 (34.57%)	22 (30.99%)		
Smoking	42 (51.85%)	39 (54.93%)	0.144	0.704
Non-smoking	39 (48.15%)	32 (45.07%)		
Alcohol	36 (44.44%)	29 (40.85%)	0.200	0.655
Non-alcohol	45 (55.56%)	42 (59.15%)		
Family history of CHD			0.654	0.419
yes	7 (8.64%)	9 (12.68%)		
no	74 (91.36%)	62 (87.32%)		
Family history of CI			0.288	0.592
yes	5 (6.17%)	3 (4.23%)		
no	76 (93.83%)	68 (95.77%)		
Vital signs				
Systolic blood pressure (mmHg)	205.14±11.44	202.34±13.93	1.359	0.176
Diastolic blood pressure (mmHg)	116.42±8.88	115.62±8.82	0.556	0.579
Heart rate (times/min)	124.49±12.91	125.41±16.57	0.382	0.703

### Hemodynamic improvements were more pronounced in the observation group

**Figure 2:**
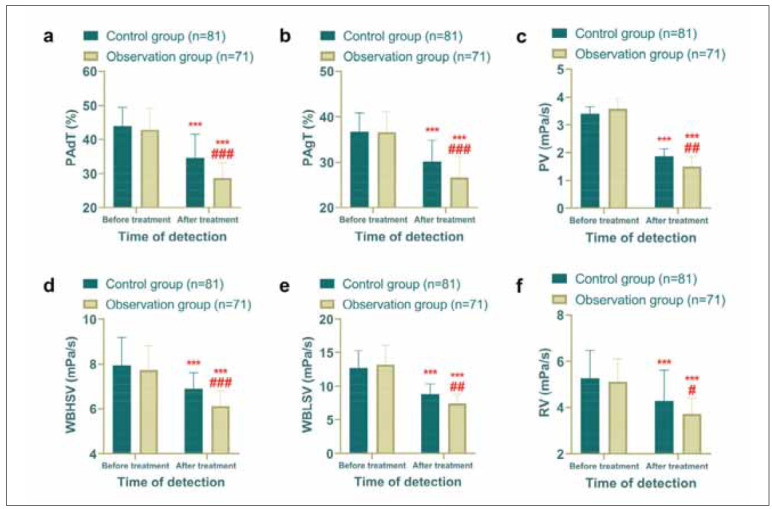
Comparison of hemodynamics, observation group had better hemodynamics after treatment. (a) Comparison of PAdT before and after treatment. (b) Comparison of PAgT before and after treatment. (c) Comparison of PV before and after treatment. (d) Comparison of WBHSV before and after treatment. (e) Comparison of WBLSV before and after treatment. (f) Comparison of RV before and after treatment. Comparison with before treatment ***P<0.001, comparison with control group *#P*<0.05, *##P*<0.01, *###P*<0.001.

Given that both CI and CHD are rooted in vascular obstruction caused by atherosclerosis [13], hemo dynamic parameters were the primary focus of this study. Post-treatment comparisons showed significant reductions in PAdT, PAgT, PV, WBHSV, WBLSV, and RV in both groups compared to the pre-treatment levels (*P*<0.05). Importantly, the observation group exhibited greater reductions in PAdT, PAgT, WBLSV, and RV than the control group (*P*<0.05), suggesting that the addition of GM to CLO therapy led to more substantial hemodynamic improvements (Figure 2).

### Neurological function showed greater improvement in the observation group

**Figure 3:**
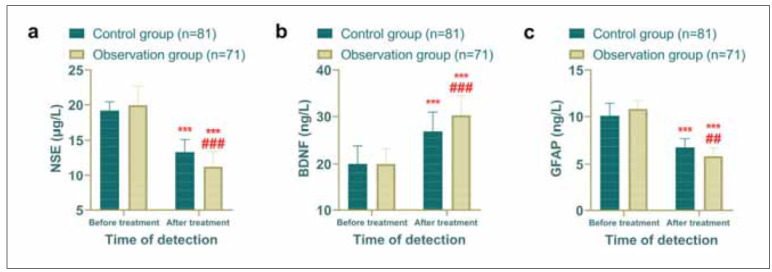
Comparison of neurocytokine, observation group had better hemodynamics after treatment. (a) Comparison of NSE before and after treatment. (b) Comparison of BDNF before and after treatment. (c) Comparison of GFAP before and after treatment. Comparison with before treatment ***P<0.001, comparison with control group *##P*<0.01, *###P*<0.001.

To evaluate neurological function, neurocytokine levels were measured in both groups. Similarly,there was no difference in the comparison of NSE, GFAP and BDNF before treatment between the two groups (p>0.05). Post-treatment results indicated that levels of NSE and GFAP decreased significantly in both groups, with the observation group demonstrating lower levels than the control group (*P*<0.05). Conversely, BDNF levels increased in both groups, with the observation group showing a more pronounced elevation compared to the control group (*P*<0.05). These findings suggest enhanced neurological recovery in the observation group (Figure 3).

### Inflammatory response was more effectively suppressed in the observation group

**Figure 4:**
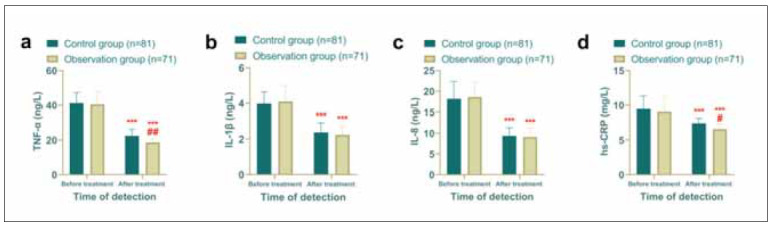
Comparison of inflammatory factors, observation group had better hemodynamics after treatment. (a) Comparison of TNF-α before and after treatment. (b) Comparison of IL-1β before and after treatment. (c) Comparison of IL-8 before and after treatment. (d) Comparison of IL-8 before and after treatment. Comparison with before treatment ***P<0.001, comparison with control group *#P*<0.05,* ##P*<0.01.

Inflammatory markers were also assessed to evaluate the systemic inflammatory response. Post-treatment levels of TNF-α, IL-1β, IL-8, and hs-CRP were significantly reduced in both groups compared to pre-treatment levels (*P*<0.05). While no significant differences were observed in IL-1β and IL-8 levels between the two groups (*P*>0.05), the observation group exhibited significantly lower levels of TNF-α and hs-CRP than the control group (*P*<0.05), indicating a more robust anti-inflammatory effect in the observation group (Figure 4).

### There was no difference in the incidence of adverse reactions between the two groups

Finally, the safety of the treatment regimens was assessed by comparing the incidence of adverse reactions. No significant differences were observed between the observation group and the control group (*P*>0.05), indicating comparable safety profiles (Table 2).

**Table 2:** Comparison of adverse reactions. There was no statistically significant difference between the two groups.

Projects	Digestive bleeding	Bloating	Hematuria	Thrombus	Muscle pain	Electrolyte disorders	Total
Control group<br>(n=81)	3 (3.70%)	4 (4.94%)	1 (1.23%)	1 (1.23%)	3 (3.70%)	2 (2.47%)	14 (17.28%)
Observation<br>group (n=71)	2 (2.82%)	3 (4.23%)	1 (1.41%)	0 (0.00%)	3 (4.23%)	1 (1.41%)	10 (14.08%)
*χ^2^*-values							0.291
*P*-values							0.589

## Discussion

This study demonstrated that the combination of GM and CLO significantly enhanced hemodynamic parameters and neurological function while effectively alleviating inflammatory responses in patients with CI complicated by CHD. These results underscore the clinical efficacy of this combined treatment regimen and offer valuable insights for optimizing the management of CI combined with CHD in the future.

As highlighted earlier, hemodynamic stability is a critical factor in the progression of both CI and CHD. The disruption of cerebral blood flow leading to localized ischemic necrosis of brain tissue, as well as coronary artery stenosis or occlusion causing myocardial ischemia, are the fundamental pathological mechanisms underlying these conditions [14]. In this study, both treatment groups exhibited significant improvements in hemodynamic parameters following the treatment, reaffirming the feasibility of both therapeutic strategies for managing CI complicated by CHD. The efficacy of CLO, a cornerstone in the clinical management of CI, has been extensively validated in prior research [15] [16]. Therefore, the observed improvements in hemodynamics in both groups were consistent with expectations. However, although no significant inter-group differences were observed in post-treatment PV and WBHSV, the observation group demonstrated further reductions in PAdT, PAgT, WBLSV, and RV. These findings suggest that the combination of GM and CLO has a more pronounced effect on improving hemodynamics in patients. In an animal study investigating lung injury and pulmonary fibrosis, Li GP et al. [17] found that ginkgolides, the primary active components of GM, exert antiplatelet aggregation effects by antagonizing the PI3K/AKT signaling pathway, thereby inhibiting thrombus formation. Based on this evidence, we propose that the combination of GM and CLO may have a synergistic effect in patients with CI and CHD, significantly enhancing cerebral blood flow and mitigating brain damage. These results align with the findings of Chen R et al. [18], who analyzed the efficacy of GM in treating CI, further corroborating our conclusions.

To further evaluate the therapeutic efficacy of GM combined with CLO in patients with CI complicated by CHD, we assessed neurocytokine levels in both groups. Neurocytokines are known to directly influence neuronal plasticity by interacting with neurons that express cytokine receptors [19]. In this study, we focused on three key neurocytokines: NSE, BDNF, and GFAP. BDNF plays a pivotal role in neuronal regeneration and the regulation of synaptic plasticity [20]. GFAP, a structural protein in the cytoskeleton, helps maintain cellular tension, and its levels rise in response to neural injury [21]. NSE, a critical enzyme in the glycolytic pathway, is elevated following braintissue damage [22]. In this study, post-treatment results showed that the observation group had lower levels of NSE and GFAP but higher levels of BDNF compared to the control group, confirming the significant neurorestorative effects of GM combined with CLO. Supporting this, Fan XX et al. [23] demonstrated in an in vitro study that ginkgolide B, a primary active component of GM, exerts profound neuroprotective effects by scavenging free radicals and mitigating oxidative stress. Furthermore, research by Chen A et al. [24] demonstrated the beneficial effects of ginkgolides on neuronal activity and functional recovery, further corroborating our findings.

Furthermore, previous evidence has confirmed the anti-inflammatory properties of ginkgolides in patients with inflammatory conditions such as Alzheimer’s disease [25]. Liu Q et al. [26] also high-lighted that the neuroprotective mechanisms of Ginkgo biloba are largely mediated through its anti-inflammatory effects. Consistent with these findings, our study measured inflammatory cytokine levels in both groups and found that post-treatment levels of TNF-α and hs-CRP were significantly lower in the observation group compared to the control group. This further supports the superior anti-inflammatory efficacy of GM combined with CLO in patients with CI and CHD. The therapeutic mechanism is likely attributed to the γ-lactone ring structure of ginkgolides, which confers a wide range of pharmacological activities, including anti-hepatotoxicity, immune stimulation, and inhibition of angiotensin release. Additionally, ginkgolides modulate the production of pro-angiogenic and anti-angiogenic factors while downregulating inflammatory cytokine levels [27]. These mechanisms are highly beneficial in alleviating the pathological progression of both CI and CHD.

Finally, the absence of significant differences in adverse reaction rates between the two groups reaf-firms the favorable safety profile of GM. As a TCM, GM has consistently demonstrated excellent safety in prior clinical studies [28] [29], further supporting its potential for combined use with CLO in future clinical applications.

Nevertheless, it is essential to address several limitations in this study. For example, the relativelysmall sample size may limit the representativeness and comprehensiveness of the findings. Furthermore, the short study duration precludes an assessment of the long-term prognostic impact of GM combined with CLO in patients with CI and CHD. Future research should aim to include larger patient cohorts, extend the follow-up period, and incorporate additional objective measures to provide a more comprehensive understanding of the therapeutic efficacy and mechanisms of GM combined with CLO.

## Conclusion

The combination of GM and CLO demonstrates significant efficacy in improving hemodynamic parameters, enhancing neuroprotective effects, and mitigating inflammatory responses in patients with CI complicated by CHD, while maintaining an excellent safety profile. This regimen represents a promising therapeutic option for the management of CI combined with CHD.

## Dodatak

### Availability of data and materials

The data used to support the findings of this study are available from the corresponding author upon request.

### Funding

No funding was received for conducting this study.

### Acknowledgements

Not applicable.

### Conflict of interest statement

All the authors declare that they have no conflict of interest in this work.
